# *Cyrtomium lacrimipinnum* (Dryopteridaceae), a new sexually reproducing fern from Phong Nha–Ke Bang National Park, Vietnam

**DOI:** 10.3897/phytokeys.275.183837

**Published:** 2026-06-01

**Authors:** Katsuhiro Yoneoka, Tao Fujiwara, Van-Son Dang, Quoc Trong Pham, Phetlasy Souladeth, Anousone Sengthong, Riko Masuda, Takenori Yamamoto, Nobuyuki Tanaka, Shuichiro Tagane

**Affiliations:** 1 Center for Frontier Research, National Institute of Genetics, 1111 Yata, Mishima, Shizuoka 411-8540, Japan Makino Herbarium, Tokyo Metropolitan University Tokyo Japan; 2 Makino Herbarium, Tokyo Metropolitan University, 1-1 Minami-Osawa, Hachioji, Tokyo 192-0397, Japan Rural Regeneration Research Center, Yamagata University Yamagata Japan; 3 Institute of Life Sciences, Vietnam Academy of Science and Technology, 85 Tran Quoc Toan Street, Xuan Hoa Ward, Ho Chi Minh, Vietnam Department of Botany, Graduate School of Science, Kyoto University Kyoto Japan; 4 Faculty of Forest Science, National University of Laos, Dongdok Campus, 01170, Xaythany District, Vientiane, Laos Center for Frontier Research, National Institute of Genetics Mishima Japan; 5 Department of Botany, Graduate School of Science, Kyoto University, Kitashirakawa-oiwake, Sakyo, Kyoto 606-8502, Japan Faculty of Forest Science, National University of Laos Vientiane Laos; 6 Rural Regeneration Research Center, Yamagata University, 1-23 Wakaba-machi, Tsuruoka, Yamagata 997-8555, Japan The Kagoshima University Museum, Kagoshima University Kagoshima Japan; 7 Department of Botany, National Museum of Nature and Science, 4-1-1 Amakubo, Tsukuba, Ibaraki 305-0005, Japan Institute of Life Sciences, Vietnam Academy of Science and Technology Ho Chi Minh Vietnam; 8 The Kagoshima University Museum, Kagoshima University, 1-21-30, Korimoto, Kagoshima, 890-0065, Japan Department of Botany, National Museum of Nature and Science Tsukuba Japan

**Keywords:** Chloroplast DNA, Laos–Vietnam border, Polypodiales, pteridophytes, Quang Tri, taxonomy

## Abstract

A new fern species, *Cyrtomium lacrimipinnum* (Dryopteridaceae), is described and illustrated from limestone ridges of Phong Nha–Ke Bang National Park, central Vietnam. It is most similar to *C. pachyphyllum* in having lateral pinnae with deeply cordate bases but is distinguished by its pinnae with acute and attenuate apices (vs. obtuse) and the presence of black or dark brown microscales on both surfaces of the pinnae (vs. confined to the abaxial surface). Spore counts of 64 per sporangium suggest a sexual reproductive mode.

## Introduction

The genus *Cyrtomium* C.Presl is a major clade within Dryopteridaceae, a family that includes some of the most species-rich lineages of extant ferns. It is a sister to *Polystichum* Roth, which together form a clade with *Phanerophlebia* C.Presl and *Pseudarachniodes* Z.Y.Zuo & Rouhan (Zuo et al. 2024). At least 40 species are currently recognized in the genus. They are distributed mainly from the tropics to warm temperate regions of the Old World (Zhang and Barrington 2013). High species diversity is concentrated in southwestern China, Vietnam, and adjacent regions, where several new species have been recently described, including *C. adenotrichum* You Nong & R.H.Jiang, *C. calcis* Liang Zhang, N.T.Lu & Li Bing Zhang, and *C. remotipinnum* Yan Liu & H.J.Wei (Lu et al. 2023; Nong et al. 2023, 2024). As of 17 February 2026, 12 species of *Cyrtomium* have been recorded in Vietnam according to GBIF.org (2026). The successive discoveries of undescribed taxa within a short period underscore the need for further exploration and taxonomic studies to better understand the diversity of *Cyrtomium* in this region.

**Figure 1.**
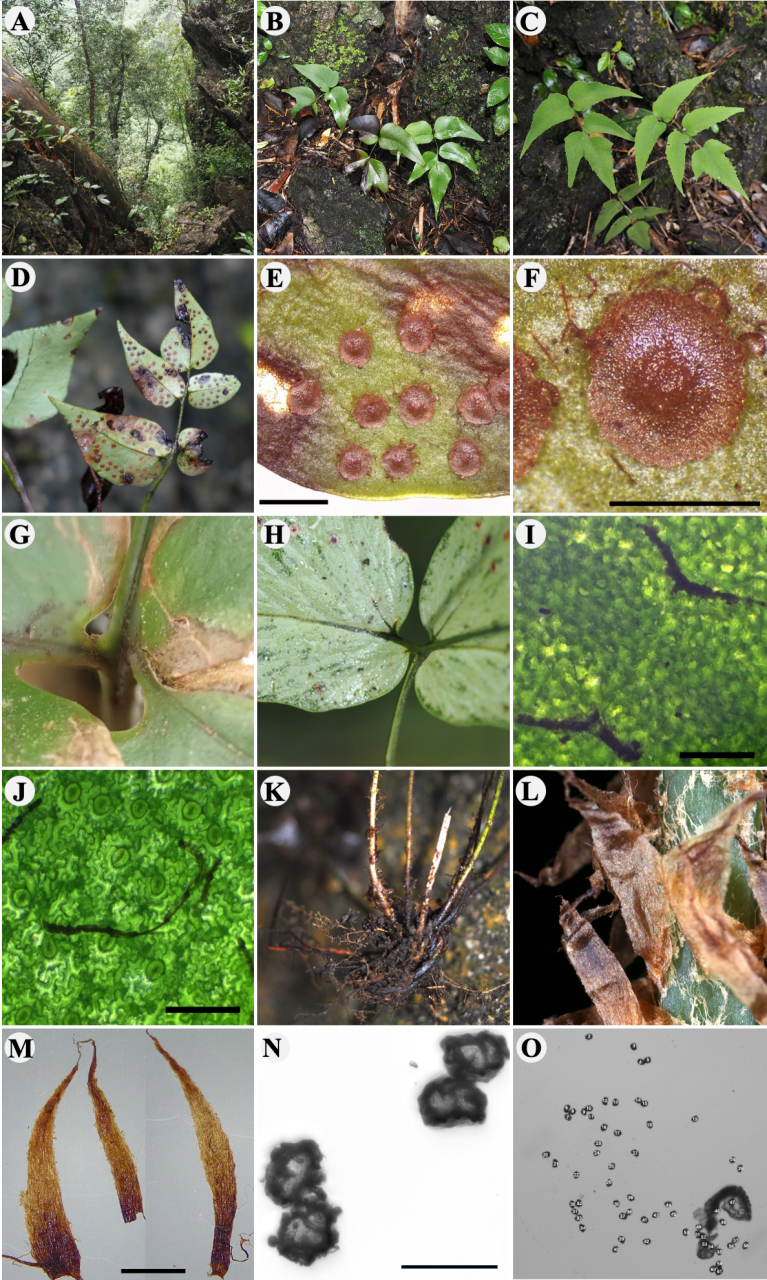
Detailed observations of *Cyrtomium lacrimipinnum*. **A**. Ecological habitat. Semi-shaded limestone area (alt. 777 m); **B, C**. Habit; **D**. Abaxial surface of a mature lamina and lateral pinnae; **E**. Sori; **F**. Indusium; **G**. Base of pinna, adaxial side; **H**. Base of pinna, abaxial side; **I**. Microscales on adaxial surface of pinna; **J**. Microscales on abaxial surface of pinna; **K**. Erect rhizome; **L, M**. Scales at stipe base; **N**. Spores; **O**. Approximately 64 spores contained in a single sporangium. Scale bars: 2 mm (**E**); 1 mm (**F**); 0.2 mm (**I**); 0.2 mm (**J**); 2 mm (**L, M**); 50 μm (**N**).

Our research group has conducted field surveys since 2023 to elucidate the flora of limestone karsts in Laos and Vietnam (e.g., Dang et al. 2025; Souladeth et al. 2025; Tagane et al. 2025). During fieldwork in Phong Nha–Ke Bang National Park, central Vietnam, in December 2024, we discovered a distinctive population of *Cyrtomium* on limestone ridges (Fig. 1A–C). This taxon shares cordate pinna bases with *Cyrtomium hemionitis* Christ and *C. pachyphyllum* (Rosenst.) C.Chr. Within *Cyrtomium*, species with cordate pinna bases are morphologically notable because most species in the genus have rounded-cuneate to truncate pinna bases. However, the newly discovered population has sharply attenuated pinna apices and hair-like microscales on both surfaces of the pinnae. Spore counts further revealed 64 spores per sporangium, indicating sexual reproduction. This combination of morphological characteristics and reproductive mode does not agree with that known in *Cyrtomium* species with cordate pinna bases, including *C. hemionitis* and *C. pachyphyllum* (Zhang and Barrington 2013). Based on this evidence, in combination with the results of molecular phylogenetic analysis, we concluded that this taxon represents a previously undescribed species of *Cyrtomium*, which is here formally described as *Cyrtomium lacrimipinnum* Yoneoka, T.Fujiw., V.S.Dang & Tagane.

## Materials and methods

### Plant sampling and herbarium survey

A total of 10 individuals of *Cyrtomium* were collected on limestone substrates in Phong Nha–Ke Bang National Park during two field expeditions in December 2024 and July 2025. Leaf tissue was dried on silica gel for DNA extraction, and vouchers were prepared from the remaining material by pressing and drying with hot air for 12 hours. These specimens were deposited in FOF, KAG, MAK, and VNM. The herbarium acronyms followed Thiers (2025, continuously updated).

In addition to our own collections, we assessed previously collected material of *Cyrtomium* from Phong Nha–Ke Bang National Park and adjacent areas. We conducted targeted searches for *Cyrtomium* specimens in the herbaria FOF, HN, KAG, MAK, and VNM to determine whether specimens attributable to the target species were present. We also examined digitized specimens online using GBIF, including records from the vascular plant collection at P and collections at MO (accessed 26 February 2026). These sources were searched to assess whether specimens with morphology similar to our collections had been recorded from other parts of Vietnam and neighboring countries.

### Morphological observations

The collected specimens were carefully examined to verify the validity of the new species, with a special focus on diagnostic characters including the size and shape of the lamina and pinnae, presence/absence of microscales, position of sori, morphology of indusia, and morphology of rhizome scales. Morphological observations were conducted using a stereomicroscope (Leica EZ4W, Leica Microsystems, Wetzlar, Germany) for gross morphology, and a light microscope (Leica DM2500, Leica Microsystems, Wetzlar, Germany) for detailed characters.

### Estimation of reproductive mode

Spore number per sporangium has been widely used as a practical indicator of reproductive mode in homosporous ferns (Grusz 2016). Sexual sporogenesis typically produces 64 spores per sporangium, whereas apogamy is often associated with a halved spore number, 32 spores per sporangium, due to modified sporogenesis (Manton 1950). This pattern has also been documented in *Cyrtomium*, including *C. falcatum* (L.f.) C.Presl (Manton 1950). To infer the likely reproductive mode of the species treated here, we counted the number of spores per sporangium using mature sporangia with clearly observable spores. We examined sporangia from two individuals collected in Phong Nha–Ke Bang National Park for which such sporangia were available. For each individual, three sporangia were selected, opened on a slide, and the number of spores per sporangium was counted under a light microscope. The counts were compared among sporangia within each individual to confirm consistency.

### Molecular experiment

Total DNA was extracted from a mature leaf (approximately 0.5 × 0.5 cm fragment) using the CTAB method of Doyle and Doyle (1987). The chloroplast *rbcL* gene (*rbcL*) and the *trnL–F* intergenic spacer sequences, including the *trnL* intron (*trnL–L–F*), were selectively amplified in polymerase chain reaction (PCR) with PrimeSTAR Max DNA Polymerase (Takara, Kyoto, Japan) under the following conditions: 95 °C for 7 min, followed by 35 cycles of 98 °C for 10 s, 58 °C for 15 s, and 72 °C for 8 s, and 72 °C for 7 min, according to Yoneoka et al. (2023). The sequences of the PCR primers used in this study are shown in Appendix 1. Raw PCR products were purified using the ExoSAP-IT Express PCR Product Cleanup Reagent (USB Products Affymetrix, Inc., Cleveland, USA) and used as templates for direct sequencing. Reaction mixtures for sequencing were prepared using the Super Dye Cycle Sequencing Kit v3.1 (Edge BioSystems, California, USA). The reaction mixtures were analyzed using an ABI 3130 Genetic Analyzer (Applied Biosystems, California, USA).

### Phylogenetic analysis

A total of 42 sequences, representing 22 taxa of *Cyrtomium* and three species of *Polystichum* as outgroups, were downloaded from GenBank and combined with newly generated sequences from this study (Appendix 2). Sequence alignment was performed using MUSCLE (Edgar 2004), and the resulting matrices were checked and edited manually in AliView (Larsson 2014). Indels were represented as gaps (-), and unavailable sequence data were coded as missing characters (?). Gaps and missing characters were retained in the aligned matrices and were not coded as additional characters for phylogenetic analyses. The aligned *rbcL* and *trnL–L–F* matrices were concatenated into a single dataset, with each region treated as an independent partition.

Maximum likelihood analysis was conducted in IQ-TREE3 (Wong et al. 2025). ModelFinder selected the GTR model for both partitions, and branch support was assessed using 1000 standard non-parametric bootstrap replicates. Bayesian inference was carried out using MrBayes v3.2.7 (Ronquist et al. 2012) under the GTR substitution model. Two independent Markov chain Monte Carlo runs, each consisting of four chains, were executed for two million generations with sampling every 1,000 generations. Convergence of the runs was assessed based on the average standard deviation of split frequencies falling below 0.01. Tracer v.1.7 (Rambaut et al. 2018) was used to evaluate the sampled trees with a focus on convergence and effective sample size (ESS), and ESS values greater than 200 were regarded as indicating adequate sampling. The first 25% of sampled trees were discarded as burn-in, and the remaining trees were used to generate a consensus tree and to calculate Bayesian posterior probabilities (PP).

## Results and discussion

Detailed morphological observations revealed that the plant we discovered differs from all previously known species of *Cyrtomium*. This species is characterized by lateral pinnae that are conspicuously cordate at the base and sharply attenuate at the apex (Fig. 1B–D) and the presence of microscales on both surfaces of the pinnae (Fig. 1I, J), by which combination it is clearly distinguished from otherwise morphologically similar taxa (Table 1).

**Table 1.** Comparison of morphological characters between *Cyrtomium lacrimipinnum* and three similar species.

Characters	*Cyrtomium hemionitis*	*Cyrtomium pachyphyllum*	*Cyrtomium grossum*	*Cyrtomium lacrimipinnum*
Laminae	simple or rarely trifoliate; deltoid-ovate	1-imparipinnate; oblong or triangular	1-imparipinnate; oblong-lanceolate	1-imparipinnate; oblong or triangular
Lateral pinnae	usually none, rarely 1 pair; deltoid; base cordate; margins entire; apex acute	deltoid-ovate, 3.5–5.0 × 2.5–3.0 cm; base cordate; margins entire; apex obtuse	oblong, 3.5–4.5 × 2.0–3.0 cm; base rounded-truncate; margins entire; apex rounded-obtuse	ovate, 3.0–9.0 × 2.0–3.5 cm; base cordate; margins entire to undulate-serrate; apex acute and attenuate
Microscales on pinnae	absent adaxially, present abaxially	absent adaxially, present abaxially	absent on both surfaces	present on both surfaces
Distribution	China (Guangxi, Guizhou, Yunnan), Vietnam (Hoa Binh, Ha Giang)	China (Guangxi, Guizhou, Yunnan)	China (Guizhou, Yunnan)	Vietnam (Quang Tri)
Reproductive mode	apomictic (triploid)	apomictic	sexual (tetraploid)	sexual

Approximately 64 spores per sporangium were observed, suggesting that this species reproduces sexually. Although the condition of the sporangia did not allow all spores to be clearly counted, the number of spores per sporangium was estimated to be 64, with up to 61 ellipsoidal spores of uniform size (35 μm in diameter) directly counted under a stereomicroscope (Fig. 1N, O). This number is clearly distinct from the 32 spores typically produced in apogamous plants and therefore supports a sexual reproductive mode based on the standard inference method using spore number (Manton 1950).

**Figure 2.**
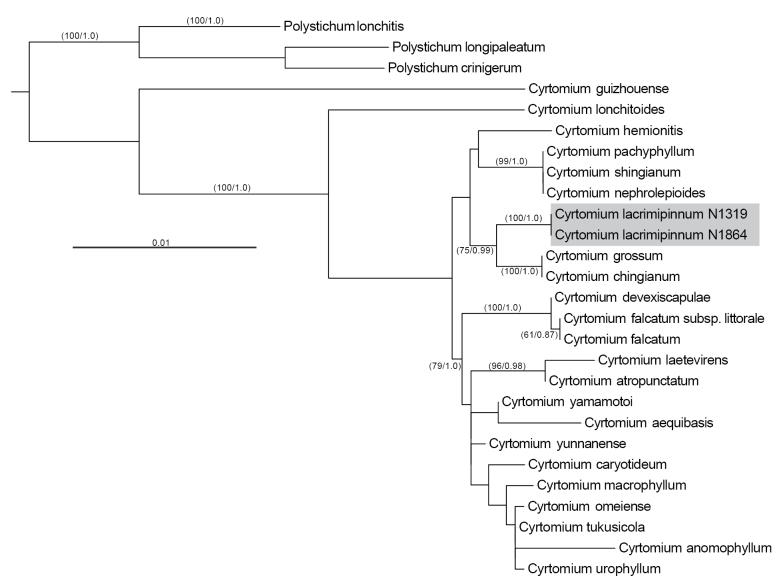
Maximum likelihood tree of *Cyrtomium* based on *rbcL* and *trnL–L–F* sequences. Support values are shown as BS/PP above the branches. The *Cyrtomium lacrimipinnum* clade is highlighted with the gray square. Scale bar: 0.01 substitutions per site.

Molecular phylogenetic analyses based on chloroplast DNA also supported the independence of the new taxon (Fig. 2). The combined dataset of *rbcL* and *trnL–L–F* comprised 2,340 bp, including 106 parsimony-informative sites. Phylogenetic trees inferred using ML and BI were congruent and placed the new species as the closest relative of *Cyrtomium grossum* Christ. However, the two taxa differed by at least 11 substitutions and were clearly separated on the phylogeny (BS = 75, PP = 0.99). These molecular results are fully consistent with the morphological comparisons. These results strongly support recognition of the discovered plants as a new species of *Cyrtomium*.

### Taxonomic treatment

#### Cyrtomium lacrimipinnum

Taxon classificationPlantaePolypodialesDryopteridaceae

Yoneoka, T.Fujiw., V.S.Dang & Tagane, sp. nov.

0916E64E-3BE9-5122-A608-EA052CD2EBA9

urn:lsid:ipni.org:names:77380804-1

Figs 1, 3

##### Diagnosis.

*Cyrtomium lacrimipinnum* is most similar to *C. pachyphyllum* in having oblong to triangular laminae with 1–5 pairs of lateral pinnae, deeply cordate pinna base, obscure venation with veinlets anastomosing to form 3–4 rows of areoles on each side of the midrib, areoles each containing 1–2 included veinlets, and dark brown stipe scales that are slightly twisted. However, it differs from *C. pachyphyllum* in having acute and attenuate pinnae apex (vs. obtuse), pinnae margins that are entire to undulate-serrate (vs. entire), and black or dark brown microscales on both surfaces of the pinnae (vs. only on abaxial surface). In addition, *C. lacrimipinnum* also resembles *C. hemionitis* in having deeply cordate pinnae base and broadly triangular terminal pinna, but distinguished by its 1–5 pairs of lateral pinnae (vs. usually simple frond or only with 1 pair in *C. hemionitis*) and the presence of black or dark brown microscales on both surfaces of the pinnae (vs. only on abaxial surface).

**Figure 3.**
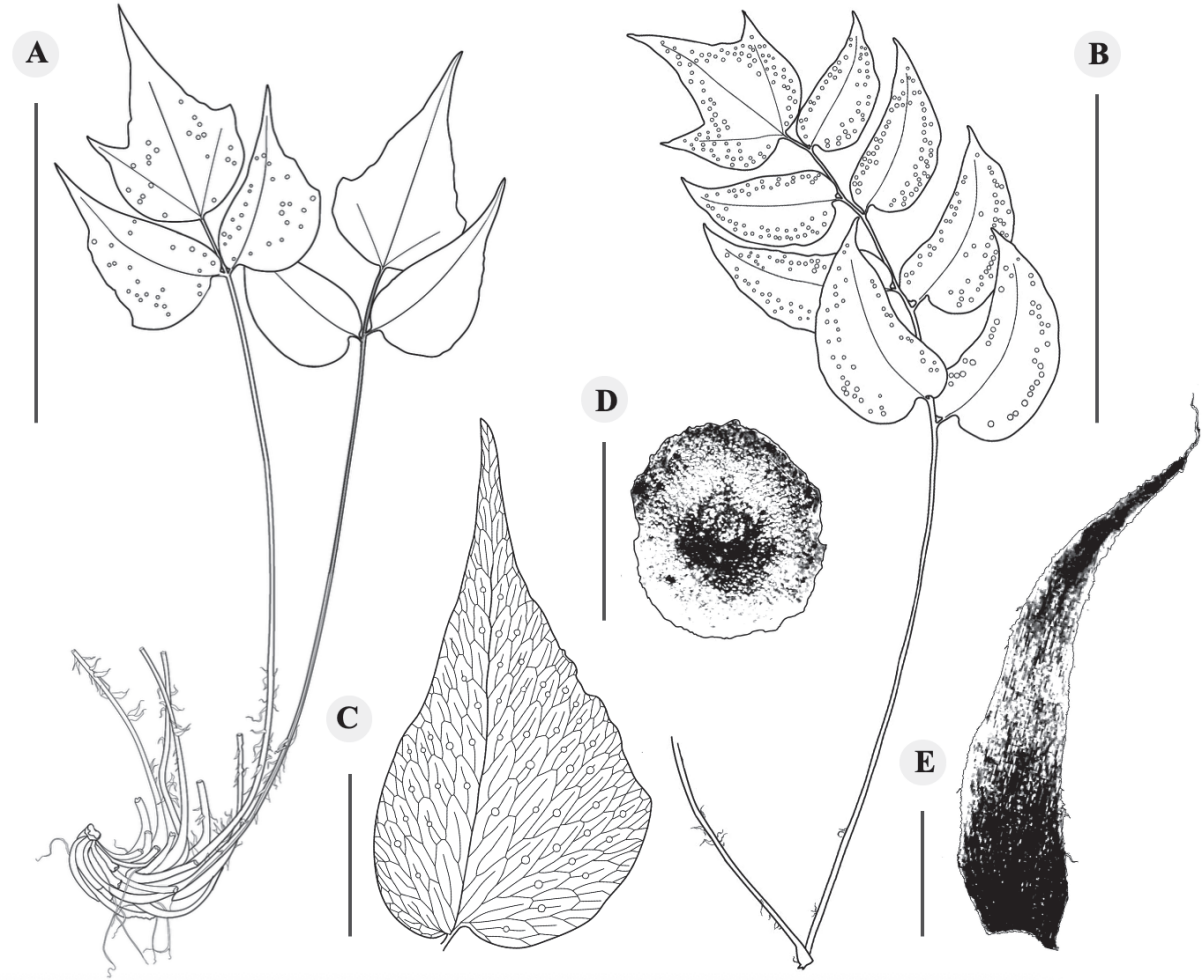
*Cyrtomium lacrimipinnum* Yoneoka, T.Fujiw., V.S.Dang & Tagane. **A, B**. Habit; **C**. Lateral pinna showing venation and sori; **D**. Indusium; **E**. Rhizome scale. Materials *Tagane et al. N1319* (KAG, MAK). Scale bars: 10 cm (**A, B**); 2 cm (**C**); 1 mm (**D**); 2 mm (**E**).

##### Type.

Vietnam • Quang Binh Province (currently Quang Tri Province): Phong Nha–Ke Bang National Park, Bach Xanh Forest, 17.40343, 106.22072, alt. 777 m, in shrubby forest on limestone ridge, 10 December 2024, *Tagane S., Dang V.S., Souladeth P., Pham Q.T., Yamamoto T., Fujiwara T., Masuda R., Souvannakhoummane K., Phengmala K. N1319* (holo-VNM [VNM00073946]; iso-KAG [KAG189432], MAK [MAK483040]).

##### Description.

***Plants*** perennial, evergreen, 13–40 cm tall. ***Rhizomes*** erect, short, densely covered with scales. ***Scales*** lanceolate, up to 8.5 mm long, dark brown, slightly twisted, apex acuminate, margin irregularly serrate, base rounded. ***Stipes*** 7–25 cm long, 1.0–3.0 mm in diam., stramineous, scaly at the basal 1/4. ***Laminae*** imparipinnate, oblong or triangular, 5–15 × 5–11 cm, coriaceous, both surfaces bearing black or dark brown microscales, apex acute to acuminate; venation pinnate, obscure on both surfaces; veinlets anastomosing to form 3–4 rows of areoles on each side of midrib, each areole with 1–2 included veinlets. ***Terminal pinna*** triangular, 4.5–5.0 × 5.0–6.0 cm, base cordate, margin usually entire or sometimes undulate-serrate. ***Lateral pinnae*** 1–5 pairs, subopposite, ascending, shortly stalked, ovate, 3.0–9.0 × 2.0–3.5 cm, base cordate, asymmetrical, apex acute or attenuate, margins entire to crenate-serrate; stalks 1.3–2.0 mm long, glabrous. ***Sori*** in 2–5 irregular rows along the margins on the abaxial surface of pinnae. Indusia 1.0–1.2 mm in diam., dark reddish brown, margin weakly dentate. ***Spores*** ca. 64 per sporangium.

##### Etymology.

The specific epithet *lacrimipinnum* combines the Latin “Lacrima,” meaning “tear,” with pinna and refers to the teardrop-like outline of the lateral pinnae.

##### Distribution.

Vietnam (currently known from Phong Nha–Ke Bang National Park and adjacent limestone areas in Quang Tri Province).

##### Habitat.

Lithophytic on semi-shaded limestone outcrops and crevices, under shrubby moist forests on ridges.

##### Additional specimen examined.

Vietnam • Quang Tri Province: Phong Nha–Ke Bang National Park, Bach Xanh Forest, 17.4039°N, 106.2209°E, alt. 765 m, in scrubland on limestone rocks, 8 July 2025, *Tagane S., Dang V.S., Souladeth P., Pham Q.T., Yamamoto T., Sengthong A., Tanaka N., Yoneoka K. N1864* (VNM [VNM00074914], KAG [KAG189926]). • Quang Binh Province (currently Quang Tri Province): Phong Nha–Ke Bang National Park, Tan Trach Commune, Bo Trach District, 17°24'24"N, 106°13'08"E, alt. ca 850 m, in a limestone area, presumed closed coniferous forest with *Calocedrus rupestris*, 37 km along Road No. 565, 13 December 2004, *Wu S.K., Phan L.K., Gong X., Xiang J.Y., Nguyen V.T., Nguyen K.S. WP1122* (MO [MO04791545]). • Quang Binh Province: Dalat Mountain, Moo Village, Thuong Hoa Commune, Minh Hoa District, 17.6714°N, 105.9228°E, alt. 550 m, 12 November 2014, *Zhang L., Zhang L., Lu N.T*. 7408 (MO [MO6871989]). • Quang Binh Province: limestone massif Ke Bang, vicinity of Yen Son Village, Thong Hoa Municipality, 72 km WNW of Dong Hoi, 17°40'N, 105°57'E, alt. 500–600 m, 16 May 1997, *L. Averyanov et al. VH 4658* (MO [MO5176107]). • Quang Bing Province: Bố Trạch, Tân Trạch, trail ca. 1 km north of Chốt Kiểm Lâm 39, Limestone forest, 26 November 2025, *Cheng-Wei Chen, Viet Dai Dang, Tich Dang, Shi-Yong Dong, Tian-Chuan Hsu, Chen Wade 7728* (SGN, TAIF).

##### Notes.

Our herbarium survey of specimens of *Cyrtomium* from Vietnam, southern China, and adjacent areas revealed three additional MO specimens from the limestone region now included in Quang Tri Province, in addition to the population collected during our fieldwork. Among them, MO6871989 differs from the holotype specimen in having undulate-serrate pinna margins. However, our field observations showed that such margins occasionally occur within populations of *C. lacrimipinnum* (Fig. 1C), and we therefore regard this difference as falling within the range of phenotypic plasticity of the species. Despite extensive examination of specimens in VNM, HN, PE, and P, we found no additional material outside the limestone region now included in Quang Tri Province, nor from neighboring regions of Laos or southwestern China. Because more than 30 individuals were observed at the type locality, the species is likely to be more widespread along inaccessible and poorly collected limestone ridges.

The inferred sexual reproductive mode of the species is also noteworthy, because sexually reproducing species are considered to be rare in *Cyrtomium*, in which most members are thought to reproduce apogamously (Ootsuki et al. 2011; Liu et al. 2012). We attempted to determine its ploidy level by counting somatic chromosomes, but no suitable material for chromosome observation was obtained. Further cytological studies will therefore be necessary to clarify the biological characteristics of this species.

### Key to species of *Cyrtomium* in Vietnam

**Table:** 

1	Laminae often simple, sometimes deeply pinnatifid near the base and forming 1 pair of lobes or free pinnae	***Cyrtomium hemionitis* Christ**
–	Laminae imparipinnate	**2**
2	Lateral pinnae cordate at base	**3**
–	Lateral pinnae rounded-cuneate, broadly cuneate, or truncate at base	**7**
3	Lateral pinnae 9 pairs or more, usually shorter than 3.5 cm	**4**
–	Lateral pinnae 7 pairs or fewer, usually longer than 5 cm	**5**
4	Laminae lanceolate-oblong; lateral pinnae 9–14 pairs, apex acute; acroscopic auricles conspicuously overlapping adjacent pinnae	***Cyrtomium calcis* Liang Zhang, N.T. Lu & Li Bing Zhang**
–	Laminae linear-lanceolate; lateral pinnae 10–26 pairs, apex rounded; acroscopic auricles separated from adjacent pinnae or close to them	***Cyrtomium nephrolepioides* (Christ) Copel**.
5	Laminae oblong-ovate, 18–24 cm long; lateral pinnae 5–7 pairs, alternate	***Cyrtomium shingianum* H.S. Kung & P.S. Wang**
–	Laminae oblong or triangular, 5–18 cm long; lateral pinnae 5 pairs or fewer, subopposite	**6**
6	Laminae covered with hairlike microscales abaxially, glabrous adaxially; apex of lateral pinnae obtuse; spores per sporangium 32	***Cyrtomium pachyphyllum* (Rosenst.) C. Chr**.
–	Laminae covered with black or dark brown microscales on both surfaces; apex of lateral pinnae acute to attenuate; spores per sporangium 64	***Cyrtomium lacrimipinnum* Yoneoka, T.Fujiw., V.S. Dang & Tagane**
7	Lateral pinnae with an acroscopic auricle	**8**
–	Lateral pinnae without an acroscopic auricle	**11**
8	Acroscopic auricles long and acute; indusia clearly dentate	**9**
–	Acroscopic auricles blunt; indusia entire or slightly incised	**10**
9	Laminae linear-lanceolate; lateral pinnae lanceolate, usually small and narrow, often shorter than 4 cm and narrower than 1.5 cm, 18–24 pairs	***Cyrtomium lonchitoides* (H. Christ) H. Christ**
–	Laminae oblong or oblong-lanceolate; lateral pinnae deltoid-lanceolate, usually longer than 9 cm, wider than 2.5 cm, 3–7 pairs	***Cyrtomium caryotideum* (Wall. ex Hook. & Grev.) C. Presl**
10	Stipe scales brown, sometimes with dark brown stripes, sparse; laminae oblong to lanceolate, papery; lateral pinnae lanceolate, base oblique, acroscopic margin subtruncate	***Cyrtomium fortunei* J. Sm**.
–	Stipe scales pale brown, dense at the base; laminae ovate-lanceolate, leathery; lateral pinnae ovate-lanceolate, base obliquely rounded-cuneate, distinctly curved acroscopically	***Cyrtomium falcatum* (L.f.) C. Presl**
11	Lateral pinnae oblong or broadly ovate, apex rounded-obtuse; pinna bases nearly equilateral	***Cyrtomium grossum* Christ**
–	Lateral pinnae oblong-ovate or lanceolate, apex acuminate, acute, or shortly caudate; pinna bases obviously inequilateral, one side subtruncate	**12**
12	Rhizome scales bicolorous, with dark brown to blackish brown centers and pale brown margins; laminae ovate-lanceolate; lateral pinnae 7–10 pairs, lanceolate, 9–15 × 2–3.5 cm	***Cyrtomium devexiscapulae* (Koidz.) Ching**
–	Rhizome scales dark brown; laminae oblong-ovate; lateral pinnae 3–8 pairs, oblong to oblong-ovate, 12–20 × 4–7 cm, basal 1–2 pairs of pinnae markedly enlarged and ovate	***Cyrtomium macrophyllum* (Makino) Tagawa**

## Supplementary Material

XML Treatment for Cyrtomium lacrimipinnum

## Appendix 1

Primers for PCR amplification and direct sequences. Target region, primer name, primer sequence, reference of primer.

*rbcL*, af3, 5’-ATGTCACCACAAACGGAGACTAAAGC-3’, Hori et al. (2018);

*rbcL*, cr3, 5’-GCGGCAGCCAATTCCGGACTCCA-3’, Hori et al. (2018);

*trnL-L-F*, FernL 1Ir1, 5’-GGYAATCCTGAGCCAAATC -3’, Li et al. (2010);

*trnL-L-F*, F, 5’-ATTTGAACTGGTGACACGAG -3’, Taberlet et al. (1991).

## Appendix 2

Information on voucher specimens and GenBank accession numbers. Taxon name, collected location, voucher, accession number of *rbcL*, and accession number of *trnL-L-F*.

***Cyrtomium aequibasis* (C.Chr.) Ching**, China, LJM049, AY694809, AY736346; ***Cyrtomium anomophyllum* (Zenker) Fraser-Jenk**. (Cyrtomium macrophyllum var. microindusium in Genbank), Japan, TNS771166, AB575110, -; ***Cyrtomium atropunctatum* Sa.Kurata** (Cyrtomium fortunei var. atropunctatum in GenBank), Japan, TNS771164, AB575105, -; ***Cyrtomium caryotideum* (Wall. ex Hook. et Grev.) C.Presl**, China, LJM026, AF537225, AY736347; ***Cyrtomium chingianum* P.S.Wang**, China, LJM032, AY694803, AY736339; ***Cyrtomium devexiscapulae* (Koidz.) Ching**, China, LJM030, AY694798, AY736334; ***Cyrtomium falcatum* (L.f.) C.Presl**, Taiwan, LJM059, AY694796, AY736332; ***Cyrtomium falcatum* (L.f.) C.Presl subsp. *littorale* S.Matsumoto ex S.Matsumoto & Ebihara**, Japan, TNS:771160, AB575104, -; ***Cyrtomium grossum* Christ**, China, LJM028, AY694805, AY736341; ***Cyrtomium guizhouense* H.S.Kung & P.S.Wang**, China, LJM029, AY694806, AY736342; ***Cyrtomium hemionitis* Christ**, China, LJM012, AY694802, AY736338; ***Cyrtomium laetevirens* (Hiyama) Nakaike**, Japan, TNS:771199, AB575108, -; ***Cyrtomium lonchitoides* (Christ) Christ**, China, LJM055, AY694800, AY736336; ***Cyrtomium lacrimipinnum* Yoneoka, T.Fujiw., V.S. Dang & Tagane**, Vietnam, N1319, LC905129, LC905131; ***Cyrtomium lacrimipinnum* Yoneoka, T.Fujiw., V.S. Dang & Tagane**, Vietnam, N1864, LC905130, LC905132; ***Cyrtomium macrophyllum* (Makino) Tagawa**, China, LJM057, AY694807, AY736343; ***Cyrtomium nephrolepioides* (Christ) Cop**., China, LJM022, AY694795, AY736331; ***Cyrtomium omeiense* Ching & Shing**, China, LJM037, AY694808, AY736344; ***Cyrtomium pachyphyllum* (Rosenst.) C.Chr**., China, X.C. Zhang 2594, EF394241, -; ***Cyrtomium shingianum* H.S.Kung & P.S.Wang**, China, LJM034, AY694804, AY736340; ***Cyrtomium tukusicola* Tagawa** (Cyrtomium macrophyllum var. tukusicola in GenBank), Japan, TNS:771200, AB575111, -; ***Cyrtomium urophyllum* Ching**, China, LJM043, AY694797, AY736333; ***Cyrtomium yamamotoi* Tagawa**, Japan, FP000209, KT831855, -; ***Cyrtomium yunnanense* Ching**, China, LHZ-f1, -, AY736345; ***Polystichum cringerum* (C. Chr.) Ching**, China, LJM062, AY694813, AY736352; ***Polystichum lonchitis* (L.) Roth**, Washington, USA, Zika18981, AF537247, AY736354; ***Polystichum longipaleatum* Christ**, China, LJM061, AY694814, AY736353.

## Additional information

### Artificial Intelligence (AI) use

The authors accept full responsibility for the content of the manuscript, including the disclosure of any use of AI.

Regarding the use of AI in the preparation of this manuscript, the authors declare the following:

Gemini 3.0 was used exclusively for English language editing and grammatical refinement of the manuscript. The scientific content and the original text were created entirely by the authors.

### Author ORCIDs

K. Yoneoka https://orcid.org/0009-0000-4985-9340

T. Fujiwara https://orcid.org/0000-0002-9397-2831

V.-S. Dang https://orcid.org/0000-0001-8681-4141

P. Souladeth https://orcid.org/0000-0001-8563-1773

R. Masuda https://orcid.org/0009-0004-4996-1109

T. Yamamoto https://orcid.org/0000-0002-5487-6652

N. Tanaka https://orcid.org/0000-0001-8703-5967

S. Tagane https://orcid.org/0000-0002-1974-7329

### Data availability

All of the data that support the findings of this study are available in the main text.
